# Entecavir plus adefovir rescue therapy for chronic hepatitis B patients after multiple treatment failures in real-life practice

**DOI:** 10.1186/1743-422X-10-162

**Published:** 2013-05-25

**Authors:** Xian-Hua Xu, Gai-Li Li, Yang Qin, Qiang Li, Fa-Qun He, Jin-Ye Li, Quan-Rong Pan, Jie-Yin Deng

**Affiliations:** 1 Department of Geriatrics, Chengdu Military General Hospital, Chengdu, 610083, People’s Republic of China

**Keywords:** Chronic hepatitis B, Multiple failures, Resistance, Combination therapy, Entecavir, Adefovir

## Abstract

**Aim:**

To evaluate the efficacy and safety of Entecavir (ETV) plus adefovir (ADV) for chronic hepatitis B (CHB) patients after multiple nucleos(t)ide analogue (NAs) failure treatment.

**Methods:**

Hepatitis B e antigen (HBeAg)-positive patients who had a suboptimal response or developed resistance to two or more previous NAs treatments were included, and all subjects were treated with ETV in combination with ADV for ≥ 24 months. Complete virologic response (CVR) was defined as an undetectability of serum hepatitis B virus (HBV) DNA level during treatment. Safety assessment was based on the increasing of serum creatinine and creatine kinase levels.

**Results:**

A total of 45 eligible patients were included. Twenty-five patients had been treated with lamivudine (LAM) or telbivudine (LdT) and developed genotypic resistance. Resistance to ADV was present in 18 patients and 4 patients had a suboptimal response to ETV. Two patients had a resistance to both LAM and ADV. The cumulative probabilities of CVR at 12 and 24 months of ETV + ADV treatment were 88.9% (40/45) and 97.8% (44/45), respectively. Although one patient failed to achieve CVR, its serum HBV DNA level decreased by 3.3 log copies/mL after 24 months of combination therapy. The cumulative probability of HBeAg seroconversion was 15.6% (7/45) and 26.7% (12/45) at 12 and 24 months of treatment, respectively. History of prior exposure to specific NAs did not make a difference to ETV + ADV treatment outcome. There were no significant adverse events related to ETV + ADV therapy observed in the study subjects.

**Conclusion:**

ETV + ADV can be used as an effective and safe rescue therapy in patients after multiple NA therapy failures, especially in the areas where tenofovir is not yet available.

## Introduction

About a quarter of the world’s population have been infected with hepatitis B virus (HBV), including 350 million patients with chronic hepatitis B (CHB); and about 15% to 25% of CHB patients would progress to life-threatening liver disease including cirrhosis and hepatocellular carcinoma [1]. Nucleos(t)ide analogues (NAs) as an important class of antiviral drugs have changed the treatment paradigm and prognosis of CHB [2]. However, the development of antiviral resistance has become a threat of NAs therapy [3,4], and an increasing number of patients experience multiple NAs treatment failures, especially when they are sequentially treated with NAs that have low genetic barrier and similar characteristics [5-7].

In past decade in China, many CHB patients have undergone sequential treatment with lamivudine (LAM), adefovir dipivoxil (ADV), telbivudine (LdT) and/or entecavir (ETV) at 1 mg to manage antiviral resistance or insufficient suppression of HBV DNA. In fact, majority of multidrug failure were developed in patients who switched to ADV monotherapy for LAM refractory, and the virological and biochemical outcomes of single ETV salvage therapy were not satisfactory [8]. And the multidrug failures or resistance have begun to emerge as an important and difficult issue for clinicians [9].

Currently, more and more evidences had showed that the development of drug resistance was associated with viral relapse, biochemical breakthrough, clinical deterioration, and even losing of favorable effects obtained by previous NAs treatment. Thus, to achieve sustained suppression of HBV DNA replication and remission of liver disease, successful management of CHB patients who developed treatment failure due to antiviral resistance or incomplete inhibition of viral replication is critical [9,10].

Recently, the increasing evidence suggests that combination therapy could effectively suppress viral replication and significantly delay or prevent the emergent of drug resistance of HBV strains, and combination therapy has become a potentially attractive therapeutic option in management of refractory CHB patients [11-16]. However, as compared to other antiretroviral therapies, the experience of combination antiviral therapy for CHB after multiple failures is relatively few at present. Recently, the combination of ETV and ADV has been widely concerned; however, its efficacy in patients who had a suboptimal response or developed resistance to two or more previous NAs treatments is not well known.

In this study, we evaluated the efficacy and safety of the combination of ETV plus ADV in CHB patients after the failure of multiple NA therapies, and the data generated in this study were anticipated to provide researchers and practitioners with more information regarding the management of CHB patients after multiple failures.

## Materials and methods

### Subjects

CHB patients with failures of two or more previous NAs therapies were treated with 0.5 mg of ETV (Bristol-Myers Squibb) plus 10 mg of ADV (GlaxoSmithKline) daily for at least 24 months. Failures of previous NAs therapies included suboptimal viral suppression (serum HBV DNA level > 10 000 copies/mL despite continued therapy for more than 1 year) or the development of resistance. Exclusion criteria were coinfection with human immunodeficiency virus or hepatitis C virus, and history of underlying renal problems. The study protocol was approved by the ethics committee of Chengdu Military General Hospital, and was conducted in accordance with the principles of the Declaration of Helsinki.

### Laboratory and clinical assessment

Serum HBV DNA, hepatitis B e antigen (HBeAg), anti-hepatitis B e antibody (Anti-HBe), alanine aminotransferase, creatinine and creatine kinase levels were detected every 3 or 6 months. Serum HBV DNA level was determined by real-time polymerase chain reaction assay(DA AN Gene Co.,Ltd., Guangzhou, China), which has a lower limit of detection at 500 copies/mL. Serum HBeAg and Anti-HBe statues were measured using Enzyme-Linked Immunosorbent Assay (Intec Stone, China). To identify mutations associated with resistance in the gene encoding HBV polymerase, PCR amplification and direct sequencing was performed.

### Definition of treatment response

The mean reduction of serum HBV DNA level was assessed during treatment. Complete virological response (CVR) was defined as a decrease of serum HBV DNA ≤500 copies/mL. The primary non-response was defined as a decrease of serum HBV DNA of less than 2 log copies/mL at 24 weeks of therapy. Viral breakthrough was defined as an increase of HBV DNA > 1 log copies/mL from nadir during ETV + ADV treatment. HBeAg seroconversion was defined as loss of HBeAg and appearance of anti-HBe on two occasions at least.

### Statistical analysis

To describe continuous variables with normal distributions, the mean ± standard deviation was used. Continuous variables without normal distributions were expressed as the median with range. Cumulative probability of CVR during the treatment period was calculated using the Kaplan-Meier method. *P* values less than 0.05 were considered statistically significant. All data were analyzed using SPSS 15.0 (Chicago, IL, United States).

## Results

### Baseline characteristics of the study subjects

A total of 52 patients with failures of two or more previous NAs therapies were screened, of which 7 patients were excluded by underlying renal problems, and the remaining 45 patients were included in the study. Detailed demographics of those 45 included patients are presented in Table 1. In this cohort, the mean age was 38.64 ± 5.82 years and 71.1% of them were male. The mean baseline serum HBV DNA level was 5.4 ± 1.3 log copies/mL. Twenty-three patients had been treated with LAM (N = 16) or telbivudine (LdT) (N = 7) and developed genotypic resistance. Resistance to ADV was present in 18 patients, and 4 patients had a suboptimal virological response to ETV. Two patients had a resistance to both LAM and ADV. The median treatment duration of ETV + ADV combination therapy was 30 months (range 23 to 38 months).

**Table 1:** The baseline demographics of patients in this study

**Characteristic**	**Value**
Gender (Male/Female, n/n)	32/13
Age, yr	38.64 ± 5.82
Serum ALT (IU/mL)	134.53 ± 32.61
Serum HBV DNA (log 10 copies/mL)	5.4 ± 1.3
LAM/LdT-resistant mutation, n(%)	23(51.11%)
rtM204I	7
rtM204I/V + rtL180M	16
ADV-resistant mutation	18(40.00%)
rtN236T	8
rtA181T/V	6
RtN236T + rtA181T/V	4
LAM + ADV resistance	2(4.44%)
Suboptimal response to ETV	4(8.89%)

### Virological response

**Figure 1:**
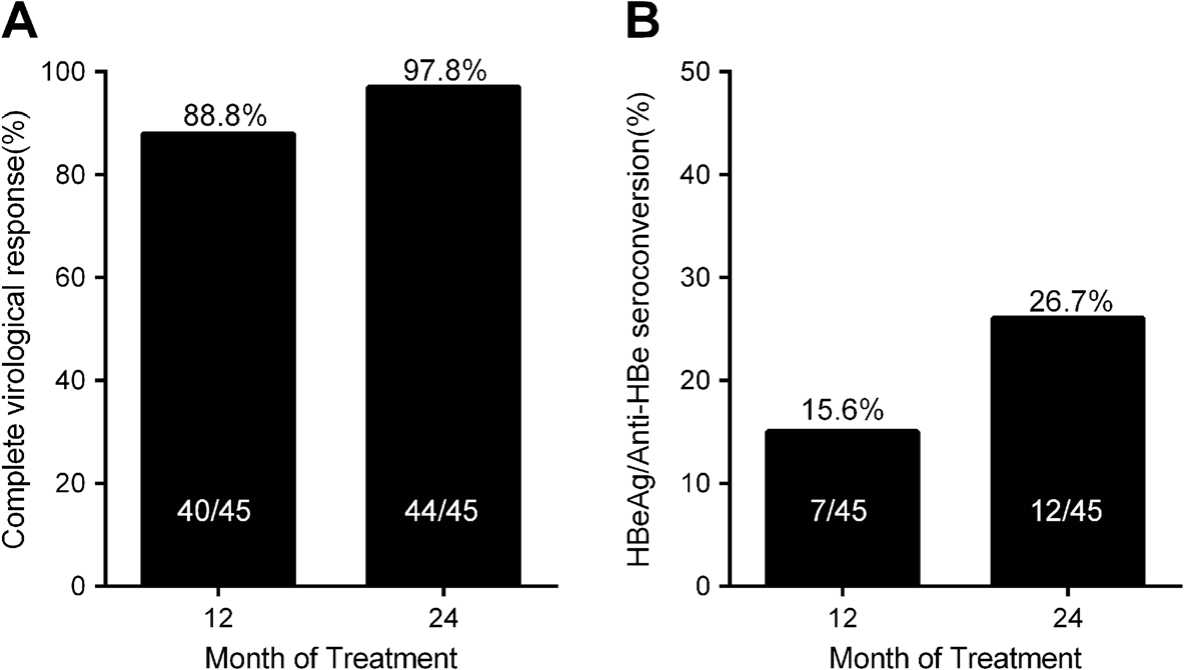
**Curative efficacy.** The percentages for complete virological response (**A**) and HBeAg/Anti-HBe seroconversion (**B**).

The probability of CVR was 88.9% (40/45) at 12 months and 97.8% (44/45) after 24 months of treatment (Figure 1A). In this study, no patient developed primary non-response or viral breakthrough during follow-up. Though one patient did not achieve CVR, its serum HBV DNA level had decreased by 3.3 log copies/mL after 24 months of combination therapy.

To define whether there is any difference in the rates of CVR according to prior exposures to different antiviral agents and genotypic resistance profile, the CVR rates were compared according to these variables using a log-rank test. There were no significant differences between patients with prior exposure to LAM and ADV *vs* LAM, ADV, and ETV (*P* =0.503). Genotypic resistance to ADV (rtA181V/T or rtN236T) and resistance to LAM/LdT did not affect CVR rates (*P* =0.876 and 0.104, respectively).

### Biochemical and serological responses

Cumulative probability of ALT normalization and HBeAg clearance was calculated with the Kaplan-Meier method. After 24 months of ETV + ADV combination therapy, all patients obtained ALT normalization; and the rate of HBeAg/Anti-HBe seroconversion was 15.6% (7/45) at 12 months and 26.7% (12/45) at 24 months of treatment (Figure 1B).

### Adverse events

No patient developed renal toxicity and creatine kinase increasing, and there were no other adverse events related to ETV or ADV therapy observed in the subjects.

## Discussion

The management of CHB has improved markedly over the past decade, primarily due to the development of effective antiviral agents. Recently, the sustained suppression of HBV DNA replication in vivo has been shown to be associated with the prevention of disease progression and complications of chronic HBV infection [1]. However, more and more evidence showed that sequential therapies with multiple NAs with low genetic barrier, significantly promoted the selection of drug-resistant strains of HBV and frequently leaded to viral breakthrough or inadequate viral response, which would inevitably not only diminish the beneficial effects of previous therapy but also limit their future therapeutic options [6,17]. Currently, NAs multiple failures are becoming a global clinical and public health problem, which is urgently needed to be solved.

A newly established concept in the management of NAs treatment failures is the superiority of add-on therapy rather than switch-to therapy [18]. However, the data on the efficacy of combination therapy for the patients with multidrug refractoriness is still limited. This study was a ‘real-world’ assessment of ETV + ADV salvage therapy in patients with developed resistance or suboptimal response to two or more previous NAs treatment. Our results demonstrated that ETV + ADV could induce ideal complete virologic response and HBeAg seroconversion, suggesting that ETV + ADV would be an effective and safe rescue therapy in CHB patients after multiple NAs therapy failures.

In this cohort, majority of the patients were exposure to sequential LAM and ADV treatment, and 9 patients had a resistance to both LAM and ADV. Though LAM + ADV has been widely used for rescue therapy in past years for those patients [5,13], patients with sequential LAM and ADV resistance had resistant mutations on the same HBV genome, which anticipated that the combination therapy of LAM + ADV might be unsatisfactory for the CHB patients with genotypic resistance associated with sequential LAM and ADV treatment. Indeed, recent data also showed that the combination therapy of LAM + ADV was not effective and was inferior to ETV containing treatment in suppressing HBV DNA [14], which indicated that ETV based combination therapy would be more superior to the LAM + ADV combination therapy in management of LAM- or ADV- resistant patients. And this superiority of ETV + ADV could be explained by the effect of ETV against ADV resistant mutation and the effect of ADV against LAM resistant mutation. In present study, we also found that ETV + ADV combination therapy showed ideal efficacy in HBV DNA suppression over 24 months in patients after LAM and ADV therapy failures, and this finding also supported the superior efficacy of ETV + ADV combination therapy in a certain degree [19].

In this study, there was one patient did not achieve CVR with ETV + ADV combination treatment. Further analysis revealed that he had a resistance to both LAM and ADV prior to ETV + ADV treatment. In fact, some previous reports had suggested that ETV + ADV combination therapy had lower efficacy in patients with both LAM and ADV genotypic resistant mutations as compared to patients with LAM single or ADV single resistant mutations [20,21].

Owing to the potent antiviral activity and high genetic barrier to resistance, ETV is now recommended as a first-line therapy for HBV infected patients in all recently published guidelines. However, there were also some patients responded poorly to ETV monotherapy [11], and the optimization therapy for ETV failure is concerned recently. In this study, there were also 4 patients with suboptimal response to ETV, and all of them had been exposure to sequential LAM and ETV treatment. As we know, there is cross-resistance site of HBV between LAM and ETV, and ETV was less effective in LAM-resistant patients, either for rtM204I or rtL180 M plus rtM204V mutants [22]. In present study, we interested found that the add-on of ADV treatment could significantly inhibit the viral replication to undetectable level for all 4 patients with suboptimal response to sequential LAM and ETV treatment.

In this study, all patients were well tolerated, and no significant adverse events related to ETV + ADV therapy observed. And the safety profile of ETV + ADV combination therapy for CHB patients in this cohort was similar to the profile reported in other studies.

Limitations of this study are the small sample size and relatively short observation duration. Thus, clinical trials with large sample size and long term follow-up are needed to confirm our findings. Because of the unavailability of tenofovir in China, we could not evaluate the efficacy of tenofovir or the combination therapy including tenofovir in for CHB patients after multiple failures, and further studies is needed to determine their superiority.

In conclusion, in CHB patients after multiple NA therapy failures, ETV + ADV can be used as an effective and safe rescue therapy, especially in the areas where tenofovir is not yet available.

## Consent

Written informed consent was obtained from the patient for publication of this report and any accompanying images.

## Abbreviations

HBV: Hepatitis B virus; CHB: Chronic hepatitis B; LAM: Lamivudine; ADV: Adefovir dipivoxil; LdT: Telbivudine; ETV: Entecavir; NAs: Nucleos(t)ide analogues; ALT: Alanine aminotransferase; HBeAg: Hepatitis B e antigen; CVR: Complete virological response.

## Competing interests

All authors declare that they have no competing interests.

## Authors’ contributions

LGL conceived the study and revised the manuscript critically for important intellectual content. XXH drafted the manuscript. XXH, QY, LQ, HFQ and LYJ made substantial contributions to its design and acquisition of data. PQR and DJY participated in the analysis and interpretation of data. All authors read and approved the final manuscript.

## References

[B1] Liaw YF Impact of therapy on the outcome of chronic hepatitis B Liver Int 2013 33 Suppl 1 111 115 23286854 10.1111/liv.12057

[B2] Yuen M-F Lai C-L Treatment of chronic hepatitis B: Evolution over two decades Journal of gastroenterology and hepatology 2011 26 Suppl 1 138 181 21199525 10.1111/j.1440-1746.2010.06545.x

[B3] Hongthanakorn C Chotiyaputta W Oberhelman K Fontana R Marrero J Licari T Lok A Virological breakthrough and resistance in patients with chronic hepatitis B receiving nucleos(t)ide analogues in clinical practice Hepatology 2011 53 1854 1917 10.1002/hep.24318 21618260

[B4] Zoulim F Locarnini S Hepatitis B virus resistance to nucleos(t)ide analogues Gastroenterology 2009 137 1593 1608 1591-1592 10.1053/j.gastro.2009.08.063 19737565

[B5] Vassiliadis T Giouleme O Koumerkeridis G Koumaras H Tziomalos K Patsiaoura K Grammatikos N Mpoumponaris A Gkisakis D Theodoropoulos K Adefovir plus lamivudine are more effective than adefovir alone in lamivudine-resistant HBeAg- chronic hepatitis B patients: a 4-year study Journal of gastroenterology and hepatology 2010 25 54 114 10.1111/j.1440-1746.2009.05952.x 19780875

[B6] Kim SS Cho SW Kim SO Hong SP Cheong JY Multidrug-resistant hepatitis B virus resulting from sequential monotherapy with lamivudine, adefovir, and entecavir: clonal evolution during lamivudine plus adefovir therapy J Med Virol 2013 85 55 64 10.1002/jmv.23440 23096938

[B7] Wang C Fan R Sun J Hou J Prevention and management of drug resistant hepatitis B virus infections J Gastroenterol Hepatol 2012 27 1432 1440 10.1111/j.1440-1746.2012.07198.x 22694205

[B8] Ryu H Lee J Ahn S Kim DY Lee M Han K-H Chon C Park J Efficacy of adefovir add-on lamivudine rescue therapy compared with switching to entecavir monotherapy in patients with lamivudine-resistant chronic hepatitis B Journal of medical virology 2010 82 1835 1877 10.1002/jmv.21898 20872709

[B9] Song ZL Cui YJ Zheng WP Teng DH Zheng H Diagnostic and therapeutic progress of multi-drug resistance with anti-HBV nucleos(t)ide analogues World J Gastroenterol 2012 18 7149 7157 10.3748/wjg.v18.i48.7149 23326119 PMC3544016

[B10] Tujios SR Lee WM New advances in chronic hepatitis B Curr Opin Gastroenterol 2012 28 193 197 10.1097/MOG.0b013e32835297ef 22450894

[B11] Kim S Cheong J Lee D Lee M Hong S Kim S-O Cho S Adefovir-based combination therapy with entecavir or lamivudine for patients with entecavir-refractory chronic hepatitis B Journal of medical virology 2012 84 18 43 10.1002/jmv.22227 22028068

[B12] Chen E-Q Wang L-C Lei J Xu L Tang H Meta-analysis: adefovir dipivoxil in combination with lamivudine in patients with lamivudine-resistant hepatitis B virus Virology journal 2009 6 163 10.1186/1743-422X-6-163 19818142 PMC2764700

[B13] Chae HB Kim MJ Seo EG Choi YH Lee HS Han JH Yoon SM Park SM Youn SJ High efficacy of adefovir and entecavir combination therapy in patients with nucleoside-refractory hepatitis B Korean J Hepatol 2012 18 75 83 10.3350/kjhep.2012.18.1.75 22511906 PMC3326991

[B14] Chen EQ Zhou TY Bai L Wang JR Yan LB Liang LB Tang H Lamivudine plus adefovir or telbivudine plus adefovir for chronic hepatitis B patients with suboptimal response to adefovir Antivir Ther 2012 17 973 979 10.3851/IMP2190 22728692

[B15] Seto WK Liu K Fung J Wong DK Yuen JC Hung IF Lai CL Yuen MF Outcome of lamivudine-resistant chronic hepatitis B after up to 5 years of combination therapy with adefovir Antivir Ther 2012 17 1255 1262 10.3851/IMP2335 22951420

[B16] Shin JW Jung SW Park BR Kim CJ Eum JB Kim BG Du Jeong I Bang SJ Park NH HBV DNA level at 24 weeks is the best predictor of virological response to adefovir add-on therapy in patients with lamivudine resistance Antivir Ther 2012 17 387 394 22293395 10.3851/IMP1945

[B17] Chen CH Wang JH Lu SN Hu TH Hung CH Chang MH Changchien CS Lee CM Treatment response and evolution of HBV resistance during lamivudine plus adefovir or entecavir therapy in patients with adefovir-resistant mutants Antivir Ther 2012 17 701 709 10.3851/IMP2074 22358132

[B18] Zoulim F Locarnini S Optimal management of chronic hepatitis B patients with treatment failure and antiviral drug resistance Liver Int 2013 33 Suppl 1 116 124 23286855 10.1111/liv.12069

[B19] Ha M Zhang G Diao S Lin M Wu J Sun L She H Shen L Huang C Shen W Huang Z Rescue therapy for lamivudine-resistant chronic hepatitis B: adefovir monotherapy, adefovir plus lamivudine or entecavir combination therapy Intern Med 2012 51 1509 1515 10.2169/internalmedicine.51.7329 22728482

[B20] Heo NY Lim YS Lee HC Chung YH Lee YS Suh DJ Lamivudine plus adefovir or entecavir for patients with chronic hepatitis B resistant to lamivudine and adefovir J Hepatol 2010 53 449 454 10.1016/j.jhep.2010.03.020 20646776

[B21] Lim YS Lee JY Lee D Shim JH Lee HC Lee YS Suh DJ Randomized trial of entecavir plus adefovir in patients with lamivudine-resistant chronic hepatitis B who show suboptimal response to lamivudine plus adefovir Antimicrob Agents Chemother 2012 56 2941 2947 10.1128/AAC.00338-12 22430972 PMC3370750

[B22] Sherman M Yurdaydin C Sollano J Silva M Liaw YF Cianciara J Boron-Kaczmarska A Martin P Goodman Z Colonno R Entecavir for treatment of lamivudine-refractory, HBeAg-positive chronic hepatitis B Gastroenterology 2006 130 2039 2049 10.1053/j.gastro.2006.04.007 16762627

